# Universal bursty behaviour in human violent conflicts

**DOI:** 10.1038/srep04773

**Published:** 2014-04-24

**Authors:** S. Picoli, M. del Castillo-Mussot, H. V. Ribeiro, E. K. Lenzi, R. S. Mendes

**Affiliations:** 1 Departamento de Física and National Institute of Science and Technology for Complex Systems, Universidade Estadual de Maringá, 87020-900 Maringá, Paraná, Brazil; 2 Instituto de Física, Universidad Nacional Autónoma de México, 04510 México D.F., Mexico

## Abstract

Understanding the mechanisms and processes underlying the dynamics of collective violence is of considerable current interest. Recent studies indicated the presence of robust patterns characterizing the size and timing of violent events in human conflicts. Since the size and timing of violent events arises as the result of a dynamical process, we explore the possibility of unifying these observations. By analyzing available catalogs on violent events in Iraq (2003–2005), Afghanistan (2008–2010) and Northern Ireland (1969–2001), we show that the inter-event time distributions (calculated for a range of minimum sizes) obeys approximately a simple scaling law which holds for more than three orders of magnitude. This robust pattern suggests a hierarchical organization in size and time providing a unified picture of the dynamics of violent conflicts.

Individual and collective violence has a large impact on society^1^,^2^,^3^–implications for health, for instance, range from psychological distress to injuries and mortality. Recent estimates of mortality from war and interpersonal violence reported values around 0.5–1 million deaths annually^1^,^2^, while the non-lethal impacts may affect millions more. In the light of the current status of violence and their impact on society, the knowledge of dynamical properties of violent conflicts is of interest not only for theoretical reasons.

Fundamental questions in the study of collective violence concern to the estimation of the number of conflict-related deaths in a given attack and how often the attacks will take place. In this direction, it has been reported a robust power-law distribution describing the number of conflict-related deaths which holds for wars, insurgent conflicts and acts of terrorism^4^,^5^,^6^,^7^. Robust patterns have also been reported for the timing of events^7^,^8^,^9^, like the evidence for heterogeneous temporal behaviour characterizing violent conflicts^7^. More recently, some of these findings have been extended to include a wide range of human confrontations^10^.

The heterogeneous temporal behaviour of violent events and the statistics of event sizes are both the result of a dynamical process. Despite this fact, sizes and times of violent events have been studied separately. Here we explore the possibility that both sizes and times in human conflicts can be characterized by a common approach. By analyzing available catalogs of violent events in distinct parts of the world, we show that the inter-event time distributions calculated for a range of values of minimum sizes can be approximately described by a single master curve which holds for more than three orders of magnitude. This result signalizes the existence of a scaling law expressing a hierarchical organization of times and sizes in violent conflicts.

## Results

**Figure 1:**
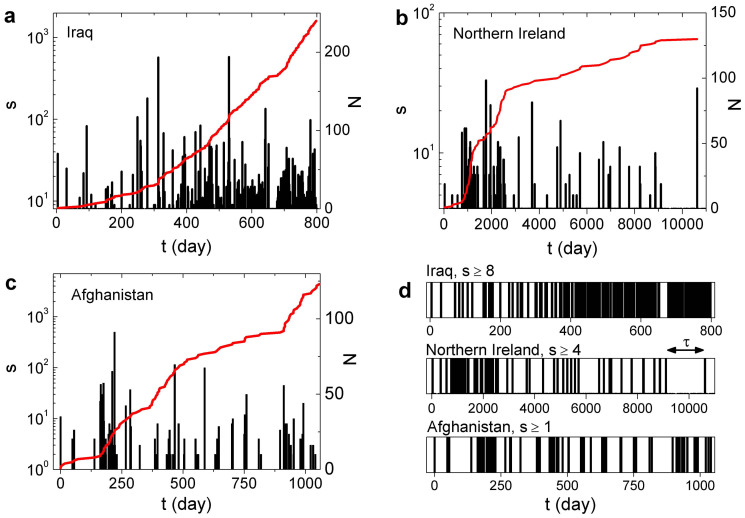
Event sizes and inter-event times. The estimated number of deaths *s* (in logarithmic scale) in a given event occurred at time *t* (in days, counted from a given initial day) for conflicts occurred in (a) Iraq, with *s* ≥ 8 in the period 2003–2005; (b) Northern Ireland, *s* ≥ 4 (1969–2001); and (c) Afghanistan, *s* ≥ 1 (2008–2010). The growth curves represent the cumulative number of events, *n*(*t*). For comparison, 〈*r*〉 = 0.30 (Iraq); 〈*r*〉 = 0.01 (Northern Ireland); and 〈*r*〉 = 0.12 (Afghanistan), where 〈*r*〉 is the mean activity rate for the given values of minimum size. (d) Inter-event times *τ* obtained from the data shown in (a), (b) and (c) for the corresponding values of minimum size.

Our findings are based in the analysis of available catalogs on violent events occurred in Iraq^7^ (2003–2005), Afghanistan^11^ (2008–2010) and Northern Ireland^12^ (1969–2001) which gives the estimated number of deaths *s* in events occurred at time *t* (for details on the datasets see Methods). We defined the inter-event time *τ* as the time interval between the beginning of two successive events, *τ_j_* = *t_j_* − *t_j_*_−1_. Here we focus on the inter-event times *τ_j_* with *j* labelling only the events with size *s* ≥ *s*_min_, where *s*_min_ is a minimum size representing a lower threshold. Figure 1 shows the empirical raw data *s* versus *t* (Fig. 1a–1c) and their correspondent inter-event times *τ* (Fig. 1d) for particular values of *s*_min_. The growth curves represent the cumulative number of events, 
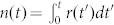
 where *r* is the activity rate defined as the number of events greater than *s*_min_ per unit of time. For a given *s*_min_ we also define the mean activity rate 〈*r*〉 which is the averaged rate over the whole time period.

**Figure 2:**
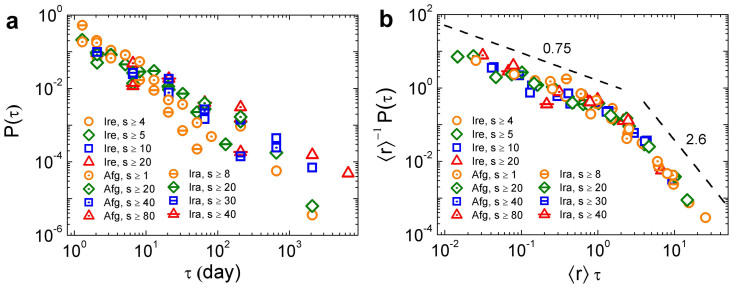
Inter-event time distributions with and without data rescaling. (a) Inter-event time distribution, *P*(*τ*), calculated for the distinct conflicts (Iraq, N. Ireland and Afghanistan) and different minimum sizes. (b) The same data, but using the scaled variables 〈*r*〉*τ* and 〈*r*〉^−1^*P*(*τ*). The dashed lines are power-law decays–with exponent 0.75 for small times and with exponent 2.6 for large times.

We then obtained *P*(*τ*), the inter-event time distribution covering events with *s* ≥ *s*_min_ (see Methods). Figure 2a shows *P*(*τ*) for the distinct conflicts and different values of *s*_min_. All curves display fat tails which can be viewed by the broad range of values of the inter-event times. As expected, the curves are scattered reflecting quantitative differences in the timing of events for distinct conflicts and minimum sizes. In order to compare the shape of the different curves we rescaled the data using the mean activity rate 〈*r*〉 and plot 〈*r*〉^−1^*P*(*τ*) versus 〈*r*〉*τ*. The rescaling causes a *s*-dependent shift of the curves since 〈*r*〉 is a function of *s*_min_. Figure 2b shows that the different curves approximately collapsed into one master curve, which holds for more than three orders of magnitude. The data collapse signalizes the existence of a scaling law in the form *P*(*τ*) = 〈*r*〉*F*(〈*r*〉*τ*), where the scaling function *F* displayed distinct behaviours for small and large values of the scaled variable. For comparison, we show a power-law decay with exponent 0.75 for small times and another power-law with exponent 2.6 for larger times (Fig. 2b).

## Discussion

The broad distributions shown in Fig. 2a reflect a large variability of the inter-event times characterizing heterogeneous temporal behaviour. This result is consistent with previous findings^7^, pointing to a scenario where high-activity intervals alternate with long low-activity periods. Bursty behaviour has also been reported for non-violent human activities and different explanations about the origin of these bursts have been proposed^13^,^14^,^15^,^16^,^17^,^18^,^19^.

The data collapse shown in Fig. 2b signalizes the existence of a scaling law governing the inter-event time distributions at distinct minimum sizes in violent conflicts. In the range considered, the dependence on the sizes are approximately governed by the mean activity rate 〈*r*〉. Since the mean activity rate is linked to the distribution of sizes, the collapse of the curves provides a unified picture of the size structure and temporal properties of the conflicts. Our findings suggest that the power-law type decay of the inter-event time distributions at small times (characterizing bursty behaviour) is just the short time limit of a general hierarchical scaling phenomenon which occurs in a wider range of time (and size) scales.

Our results contain some elements of similarity with previous findings in natural phenomena. For example, it has been shown that the inter-event time distributions for earthquakes whose magnitudes are greater than a lower threshold (taken over different spatial windows) collapse onto a single curve, providing a basis for the proposed unified scaling law^20^. This general scaling phenomenon captures elements of the Gutenberg-Richter law^21^,^22^ and the Omori law^23^ for earthquakes unifying these observations. A similar scaling behaviour for the inter-event time distribution has been reported in fracture experiments, even in the absence of spatial degrees of freedom^24^. The data collapse shown in Fig. 2b for violent conflicts is remarkably similar those obtained in earthquakes and fracture experiments^20^,^24^. Specifically, the shape of the scaling function *F* and even the reported values of the power-law exponents for small and large times are in good agreement with the results shown in Fig. 2b for human conflicts.

Exponents around 1 characterizing the inter-event time distribution at small times arises in seismic-like phenomena as a consequence of the Omori law, while exponents around 2.5 at large inter-event times have been explained in terms of temporal variations of the activity rate^20^,^24^. The power-law exponent ≈0.75 for small times (Fig. 2b) does not necessarily imply an underlying Omori law governing the timing of violent conflicts but points to this possibility. In addition, the power law exponent ≈2.6 for large times (Fig. 2b) could be related to temporal variations of the activity rate of violent events–which can be seem in the non-linear behaviour of the growth curves shown in Figs. 1a–1c.

Despite the fact that human activities and natural phenomena are very different in nature, it has been suggested that both could be described by a common approach^5^,^25^. For example, the occurrence of earthquakes has been related to the relaxation of accumulated stress after reaching a threshold as in self-organized criticality (SOC)^20^,^26^. Analogously, violent events in human conflicts could be associated with a threshold mechanism. In this scenario, a description of human conflicts in terms of SOC seems plausible. Our findings are consistent with this possibility, providing quantitative support for the analogy between patterns in human conflicts and natural phenomena exhibiting SOC.

## Methods

### Data source and processing

To study the timing of violent conflicts we select three datasets containing time-stamped records–(a) Iraq; (b) Afghanistan; and (c) Northern Ireland datasets. Iraq data were obtained from the supplementary information of Ref. 7, covering 3000 events during 803 days within 2003–2005. Each record gives the size of the event (the estimated number of deaths) and the day that the event occurred or the day that the event started, in the case of multi-day attacks. Afghanistan data, from ref. 11, consist of media-reported civilian deaths occurred in a period of 1056 days within January 2008–December 2010–a total of 123 events containing at least one killed. Northern Ireland data are based on Ref. 12, a dataset covering 11,839 days within July 1969–December 2001. Unique events were obtained by aggregating single deaths by day, giving 2143 events in the period considered. We consider all deaths in each dataset, independently of the group that performed the attack (e.g. non-state groups or state security organizations).

### Inter-event time distributions

For each dataset, we obtained the consecutive inter-event times at a given *s*_min _and removed null time intervals (*τ* = 0) in order to study temporally separated events. The distributions *P*(*τ*) for different conflicts and values of *s*_min_ correspond to the probability density function (pdf) of the data. The number of bins was computed based on the length of data and the binning procedure was applied on log-transformed data.

## Author Contributions

S.P., M.C.-M. and R.S.M. designed the research. S.P. and R.S.M. analyzed the data and wrote the main manuscript text. H.V.R. and E.K.L. contributed to the preparation of the figures. All authors reviewed the manuscript.
